# DEAD-box helicase 27 enhances stem cell-like properties with poor prognosis in breast cancer

**DOI:** 10.1186/s12967-021-03011-0

**Published:** 2021-08-06

**Authors:** Shan Li, Jinfei Ma, Ang Zheng, Xinyue Song, Si Chen, Feng Jin

**Affiliations:** 1 Department of Breast Surgery, The First Affiliated Hospital of China Medical University, Shenyang, Liaoning Province China; 2 Department of Pharmacology, School of Pharmacy, China Medical University, Shenyang, Liaoning Province China; 3 Department of Microbial and Biochemical Pharmacy, School of Pharmacy, China Medical University, Shenyang, Liaoning Province China

**Keywords:** DEAD-box helicase 27 (DDX27), Breast cancer, Stem cell-like properties, Prognosis

## Abstract

**Background:**

Although the rapid development of diagnosis and treatment has improved prognosis in early breast cancer, challenges from different therapeutic response remain due to breast cancer heterogeneity. DEAD-box helicase 27 (DDX27) had been proved to influence ribosome biogenesis and identified as a promoter in gastric and colorectal cancer associated with stem cell-like properties, while the impact of DDX27 on breast cancer prognosis and biological functions is unclear. We aimed to explore the influence of DDX27 on stem cell-like properties and prognosis in breast cancer.

**Methods:**

The expression of DDX27 was evaluated in 24 pairs of fresh breast cancer and normal tissue by western blot. We conducted Immunohistochemical (IHC) staining in paraffin sections of 165 breast cancer patients to analyze the expression of DDX27 and its correlation to stemness biomarker. The Cancer Genome Atlas-Breast Cancer (TCGA-BRCA) database and the Clinical Proteomic Tumor Analysis Consortium (CPTAC) database were used to analyze the expression of DDX27 in breast cancer. Kaplan–Meier survival analysis were used to investigate the implication of DDX27 on breast cancer prognosis. Western blot, CCK-8 assay, Transwell assay and wound-healing assay were carried out to clarify the regulation of DDX27 on stem cell-like properties in breast cancer cells. Gene Set Enrichment Analysis (GSEA) was performed to analyze the potential molecular mechanisms of DDX27 in breast cancer.

**Results:**

DDX27 was significantly high expressed in breast cancer compared with normal tissue. High expression of DDX27 was related to larger tumor size (*p* = 0.0005), positive lymph nodes (*p* = 0.0008), higher histological grade (*p* = 0.0040), higher ki-67 (*p* = 0.0063) and later TNM stage (*p* < 0.0001). Patients with high DDX27 expression turned out a worse prognosis on overall survival (OS, *p* = 0.0087) and disease-free survival (DFS, *p* = 0.0235). Overexpression of DDX27 could enhance the expression of biomarkers related to stemness and promote stem cell-like activities such as proliferation and migration in breast cancer cells.

**Conclusion:**

DDX27 can enhance stem cell-like properties and cause poor prognosis in breast cancer, also may be expected to become a potential biomarker for breast cancer therapy.

**Supplementary Information:**

The online version contains supplementary material available at 10.1186/s12967-021-03011-0.

## Background

Breast cancer has already surpassed lung cancer and developed into the major malignant tumor in women all around the world with 2.3 million new cases in 2020 based on GLOBOCAN 2020 [1]. Although the rapid development of diagnosis and treatment has improved prognosis in early breast cancer, challenges from different therapeutic response remain due to breast cancer heterogeneity.

DEAD-box helicase 27 (DDX27) pertains to the DEAD-box RNA helicases family, which is a classical ATP-dependent helicases family containing conserved D-E-A-D (Asp-Glu-Ala-Asp) sequences. This family has been confirmed to participate in various processes containing RNA transportation, RNA degradation, glucose metabolism, lipid metabolism, ribosome biosynthesis, tumorigenesis, cancer development and so on [2–8]. DDX27 was proved to take part in the processes of ribosome biogenesis, which performed an important function in cell proliferating. DDX27 could regulate the 47S ribosome RNA formation and associated with PeBow-complex independently [9]. In the process of skeletal muscle myogenesis, DDX27 was reported to influence ribosome RNA maturation, ribosome biogenesis and specific transcription [10]. DDX27 was also related to the development of malignant tumor. DDX27 had been confirmed to promote the development and metastasis in hepatocellular carcinoma and gastrointestinal cancer with poor prognosis [11–13]. Until now, the status of DDX27 expression and its implication on breast cancer remains unclear.

Breast cancer stem cells refer to a fraction of cells with strong self-renewal capacity and a huge potential of multiple differentiation in breast cancer, which can promote the courses of tumorigenesis, development, metastasis and drug resistance [14–16]. Studies proved that members of DEAD-box RNA helicases family could affect the biological behavior of cancer stem cells in various cancers [17–19]. The only member of DEAD-box RNA helicases family which has exactly effects on breast cancer stem cells is DDX17, who can enhance stem cell-like activities by combining with SOX2 or mechanism of promoting stem-like properties under hypoxia [20, 21]. DDX27 was reported to promote stem cell-like characteristics and cause poor prognosis in gastric and colorectal cancer [11, 22], while the influence on stemness in breast cancer remains unclear. Hence, it is of great significance to explore the influence of DDX27 on tumorigenesis, progress and the association with stem cell-like properties in breast cancer, which might suggest a new idea for diagnosis and therapy.

In our study, DDX27 was highly expressed in both bioinformatic analysis and samples from breast cancer patients. High expression of DDX27 was firstly reported in breast cancer with the close connection to clinicopathological factors and caused a shorter survival. The expression of DDX27 was positively related to stemness biomarkers and promoted the stem cell-like activities. Furthermore, Gene Set Enrichment Analysis (GSEA) suggested that DDX27 might have an effect on breast cancer by various ways. Hence, DDX27 can enhance the stem cell-like properties meanwhile leading to a poor prognosis in breast cancer, which means DDX27 may become a potentially significant prognosis biomarker and therapeutic target.

## Methods

### Bioinformatic analysis of DDX27 expression in breast cancer

Gene expression data used for bioinformatic analysis were downloaded from The Cancer Genome Atlas-Breast Cancer (TCGA-BRCA) database (https://cancergenome.nih.gov/), which totally contained 1109 breast cancer and 113 normal samples. Integration and normalization of all data were performed by edgR package. After optimized the data from the same patients or formalin fixed samples, we finally obtained 1071 breast cancer samples and 113 normal samples for this analysis. The expression of DDX27 was analyzed between the 1071 breast cancer samples and 113 normal samples. Also, it was analyzed between the 113 normal samples and the matched breast cancer samples from the same patients. Further, the expression of DDX27 in breast cancer samples was extracted and classified into high and low expression by median value for the subsequent studies. UALCAN (http://ualcan.path.uab.edu/index.html) is an online analysis site of public oncology database. We used UALCAN to analyze the protein expression of DDX27 in breast cancer and normal tissue on the basis of Clinical Proteomic Tumor Analysis Consortium (CPTAC) database.

### Kaplan–Meier survival analysis and gene set enrichment analysis

Kaplan–Meier Plotter (http://kmplot.com/analysis/) is an online database containing survival information and gene expression of various cancers [23]. This website was used to analyze the implication of different DDX27 expression on overall survival (OS) in the total population of breast cancer patients and the patients with different molecular subtypes. The cutoff value of high and low DDX27 expression was split by the website automatically and the results were shown by Logrank *p* value. Gene Set Enrichment Analysis (GSEA) software 4.0.3 was performed to analyze the potential mechanism related to DDX27. Gene expression file for GSEA was the integrated and normalized data from BRCA-TCGA which were classified by DDX27 expression as we mentioned before. Another input file was the phenotype label contained ‘high’ and ‘low’ expression. Gene sets used in this analysis were downloaded from the Molecular Signatures Database (MsigDB). In order to ensure the creditability of the analysis results, we chose 1000 permutations in the software. Pathways which were significantly relevant to DDX27 were chosen according to the normalized enrichment score (NES) meanwhile filtered by normalized *p* < 0.05 and false discovery rate (FDR) < 25%.

### Patients and tissue samples

Under the permission from the Ethics Review Committee in the First Affiliated Hospital of China Medical University (AF-SOP-07-1.1-01), fresh cancer tissue with paired adjacent normal tissue (n = 24) and paraffin-embedded specimens (n = 165) were obtained. All included patients were pathologically diagnosed as invasive ductal carcinoma. None of the patients received breast-conserving surgery or neoadjuvant therapy. Twenty-four pairs of fresh specimens were collected within 30 min after surgery and 165 paraffin-embedded specimens for IHC were collected during Jan 2014–Dec 2015. Clinical and pathological information including age, tumor size, lymph nodes status, estrogen receptor (ER) status, progesterone receptor (PR) status, human epidermal growth factor receptor 2 (HER2) status, histological grade, ki-67 index, molecular subtypes and TNM stage was obtained from the Hospital Information System.

### Western blot

Samples of tissue and cells were lysed by the compound of RIPA buffer and protease inhibitors (Sigma-Aldrich) for half an hour on ice after washed by phosphate-buffered saline (PBS) twice. Then the samples were centrifuged for 15 min in the condition of 14,000×*g* and 4 °C. Protein from tissue and cells was quantified by BCA assay kit (Beyotime, Jiangsu, China). Protein with equal quantity was transferred onto the polyvinylidene fluoride (PVDF) membranes (Millipore, Bedford, MA) after electrophoresed by SDS-PAGE. The membrane was sealed with bovine serum albumin (BSA) and hatched with primary antibodies at 4 °C overnight, then the secondary antibodies at room temperature for one hour. The primary antibodies for experiments contained anti-DDX27 (1:2000, Abcam, USA), anti-SOX2 (1:1000, Proteintech, China), anti-OCT4 (1:1000, Cell Signaling Technology, USA) and β-actin (1:2000, Proteintech, China). Membranes were tested with an enhanced chemiluminescence detection kit (BOSTER, USA).

### Immunohistochemical staining

Each paraffin-embedded sample was cut into 4 μm sections, dewaxed routinely, and dehydrated with gradient ethanol. All sections were retrieved antigen in citrate buffer with high pressure. The sections were restored to room temperature and blocked the activity of endogenous peroxidase by 3% hydrogen peroxide. Then, all the slides were incubated with anti-DDX27 (1:2000, Novus Biologicals, USA) or anti-OCT4 (1:500, Cell Signaling Technology, USA) at 4 °C overnight. On the second day, the sections were incubated with the secondary antibody at room temperature for one hour after washed by PBS. Sections were stained by diaminobenzidine (DAB) and counterstained by hematoxylin. Finally, slides were observed and captured with microscope.

### Evaluation of IHC

Sections with DAB staining were evaluated blinded by two pathologists separately. The expression of DDX27 and OCT4 was assessed on the basis of the intensity of staining and the ration of positive stained cells. The intensity was divided into deep (3), medium (2), light (1) and negative (0). The positive stained cells were scored as follow: 0 (0–5%), 1 (6–25%), 2 (26–50%), 3 (51–75%), 4 (76–100%). Final score of IHC staining was given by multiplication of percentage score and intensity. In this way, 165 patients were segmented into different groups in accordance with the expression of DDX27 and OCT4: high expression (final score ≥ 4) and low expression (final score ≤ 3).

### Cell lines and culture

Breast cancer cell lines MCF-7 and T47D gained from the American Type Culture Collection (ATCC) were cultured by high-glucose (4.5 mg/ml) DMEM (HyClone, USA) with 10% serum (Tianjin Hao Yang Biological Manufacture CL, China). MCF-7 mammosphere (MCF-7 MS) and T47D mammosphere (T47D MS) were inducted and cultured by DMEM/F12 (Gibco) with EGF 20 μg/L (Promega), b-FGF 10 μg/L (Promega) and 2% B27 (Gibco). All of the cells used in our research were cultured in the condition containing 5% CO_2_ and 95% air at 37 °C.

### Cell transfection

MCF-7 and T47D cells were transferred with over-expression and negative control plasmid (Genechem, Shanghai, China) using Lipofectamine 3000 (Thermo, USA).

### Wound-healing assay

DDX27 over-expression and negative control plasmid were transfected into cells until 70% confluency in six-well plates. Linear “scratches” were created in straight lines with sterile tips. Then, cells were added serum-free medium after washing with PBS three times and photographed by microscope at 0 h, 24 h, 48 h and 72 h. The area of wound-healing was analyzed by Image J.

### Transwell assay

MCF-7 and T47D transfected with DDX27 over-expression and negative control plasmid were starved with serum-free medium for 4 h. 100 μL serum-free medium with 20,000 cells and 600 μL medium with 10% serum were added into the upper and lower chamber separately. All the cells migrated to the lower chamber were fixed and stained by paraformaldehyde and crystal violet 48 h later. Three random fields were counted by Image J software for each chamber.

### Cell counting kit-8 assay

Cell Counting Kit-8 (CCK-8) assay (Dojindo, Kumomoto, Japan) was performed to test the proliferation capability of cells. Cells transfected with DDX27 over-expression and negative control plasmid were seeded in 96-well plates with 2000 cells in each well. After 24 h, 48 h and 72 h of transfection, we added 10μL CCK-8 solution in each well and then put the plate back into incubator for 2 h. Finally, we used Anthos 2010 Microplate Reader (Anthos Labtec Instruments GmbH, Austria) to detect the OD value at 450 nm.

### Statistical analysis

In this study, GraphPad Prism 8.0 (La Jolla, CA, USA) and SPSS 24.0 (Chicago, IL, USA) were performed to analyze the statistics. Results of our experiments were shown as the mean ± standard deviation (SD) for three times independently. Analysis of two groups were implemented by Student’s independent t test. Pearson chi-square test, Logistic regression and Fisher’s exact test were carried out to access the correlation of DDX27 and clinicopathological factors. The relevance between DDX27 and OCT4 was calculated by Spearman correlation analysis. The probabilities of survival were assessed by Kaplan–Meier assay. The condition of OS and DFS were assessed by Cox regression. Statistically significant probability values were defined as ≤ 0.05.

## Results

### Expression of DDX27 in breast cancer

**Fig. 1:**
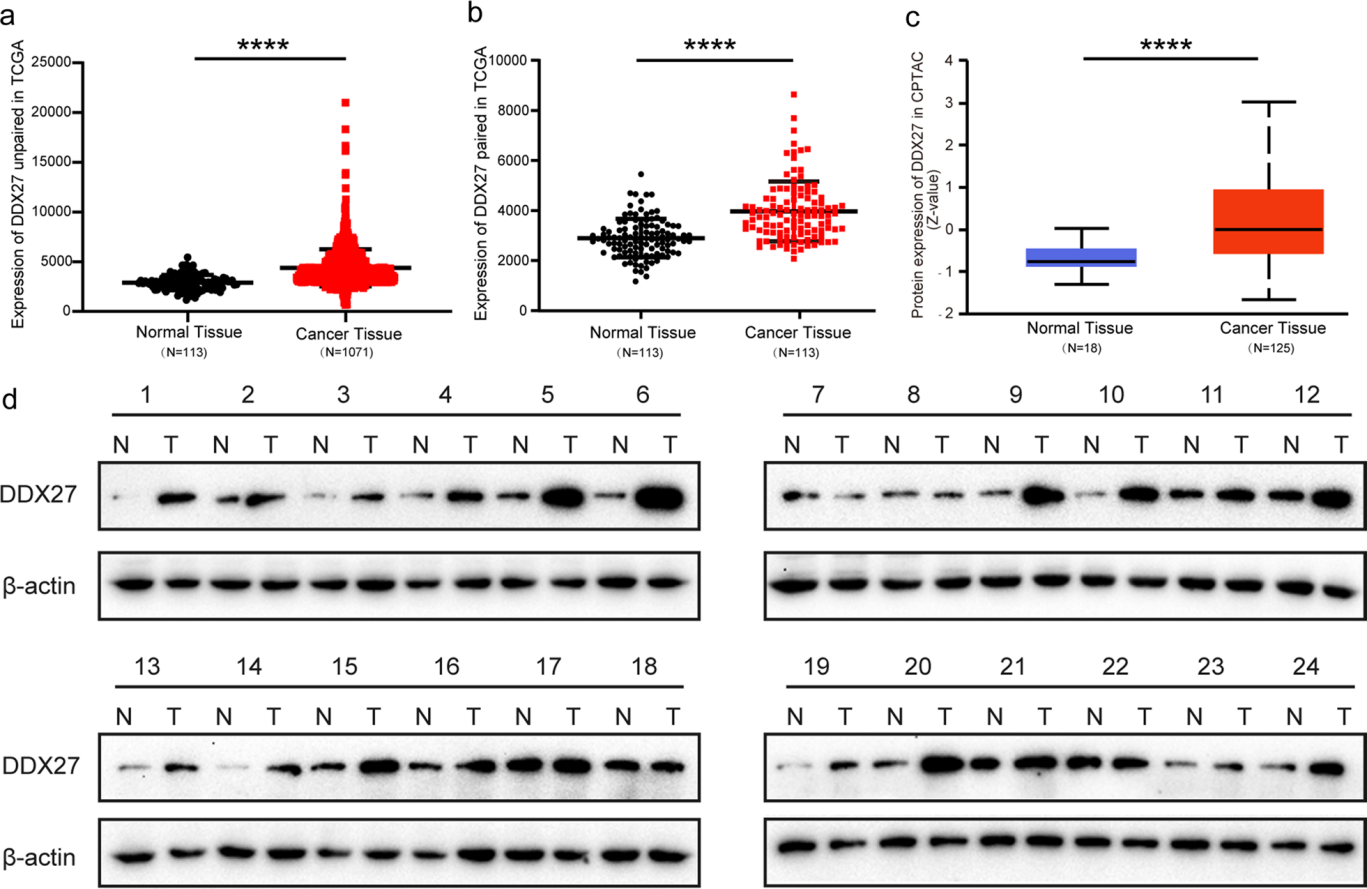
Expression of DDX27 in breast cancer. **a** DDX27 was significantly high-expressed in breast cancer than normal tissue in unpair analysis based on TCGA-BRCA database (t = 14.713 and *p* < 0.0001). **b** DDX27 was significantly high-expressed in breast cancer than normal tissue in paired analysis based on TCGA-BRCA database (t = 7.804 and *p* < 0.0001). **c** Protein expression of DDX27 was significantly higher in breast cancer than normal tissue based on CPTAC database (*p* < 0.0001). **d** DDX27 was high-expressed in fresh breast cancer tissue compared with matched normal tissue (n = 24, *p* < 0.0001). *p < 0.05, **p < 0.01, ***p < 0.001, and ****p < 0.0001

**Fig. 2:**
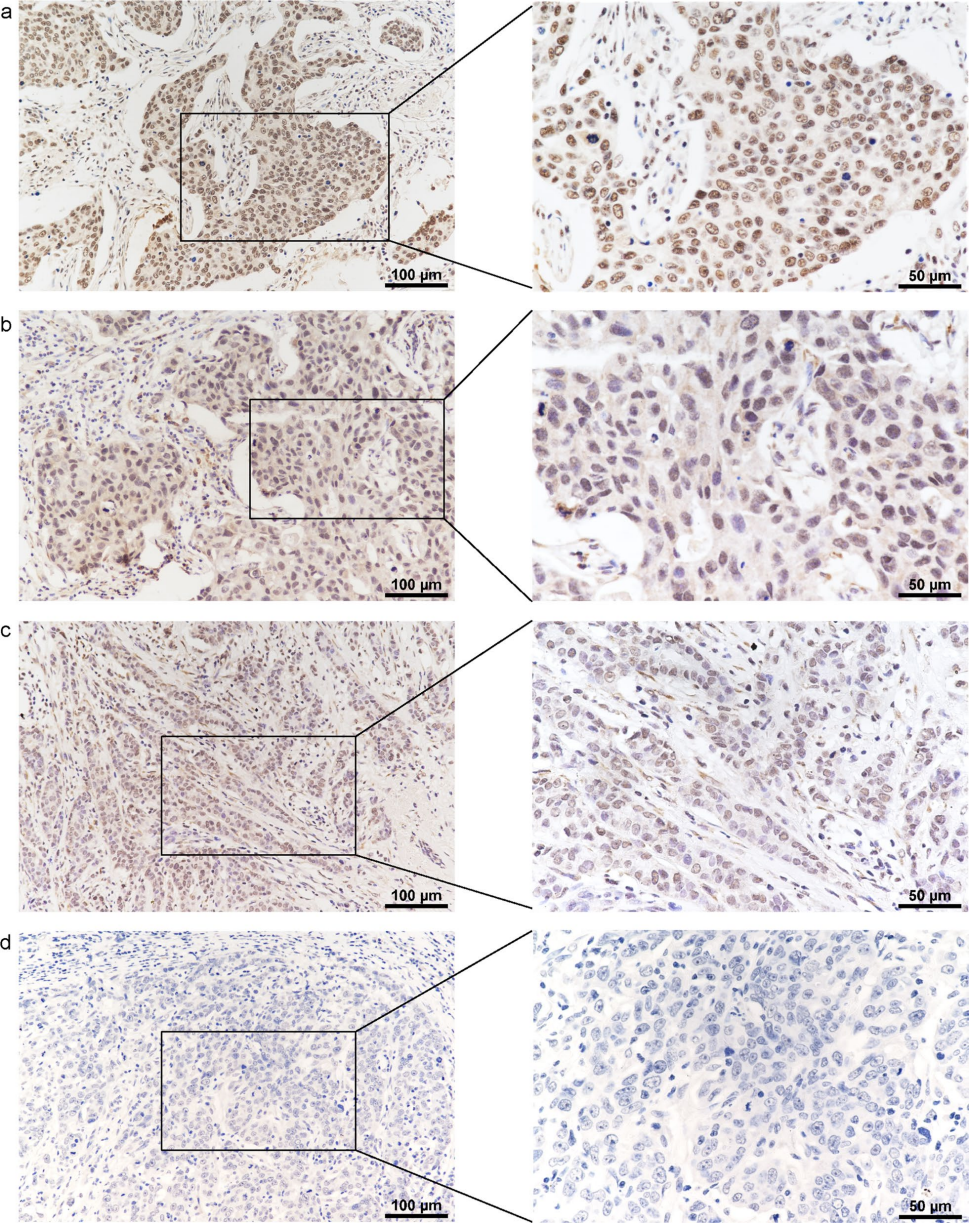
IHC staining of DDX27. **a** Deep staining; **b** Medium staining; **c** Light staining; **d** Negative staining

Analysis based on TCGA-BRCA database showed that DDX27 was significantly high expressed in cancer whether it's a paired analysis or not (*p* < 0.0001, Fig. 1a, b). Results of the UALCAN website also proved that the protein expression of DDX27 in CPTAC database was significantly higher in breast cancer than in normal samples (*p* < 0.0001, Fig. 1c). We evaluated DDX27 expression in 24 pairs of breast cancer samples via western blot assay and confirmed that DDX27 was significantly high expressed in cancer than matched normal tissue (*p* < 0.0001, Fig. 1d). Then, we performed IHC to assess the expression of DDX27 in 165 breast cancer patients and found DDX27 was expressed in nucleus (Fig. 2a–d). As a result, DDX27 was significantly highly expressed in cancer comparing to the normal breast tissue (11/40, 27.5%) and there were 97 breast cancer samples (58.8%) with high-expressed DDX27 and 68 samples (41.2%) with low expression.

### Association of DDX27 expression with clinicopathological factors and prognosis

Association between DDX27 and clinicopathological characteristics were performed to access the influence of DDX27 in breast cancer patients. Univariate analysis (Table 1) suggested that DDX27 was positively associated with larger tumor size (*p* = 0.0005), positive lymph nodes (*p* = 0.0008), higher histological grade (*p* = 0.0040), higher ki-67 (*p* = 0.0063) and later TNM stage (*p* < 0.0001) in 165 breast cancer patients. Analysis of UALCAN showed that high expression of DDX27 was significantly related to clinical stage and molecular subtypes (Additional file 1: Figure S1a, b), which had the consistent result with our analysis in clinical stage but not in the molecular subtypes.

**Table 1:** The relationship between DDX27 expression and clinical pathology factors in 165 breast cancer patients

Factors	Number (%)	DDX27		χ^2^	*p*-value	Crude OR (95% CI)
		Low (%)	High (%)			
Age				0.253	0.6149^a^	
< 60	127 (76.97)	51 (30.91)	76 (46.06)			Reference
≥ 60	38 (23.03)	17 (10.30)	21 (12.73)		0.6151^b^	0.829 (0.399–1.722)
Tumor size				12.053	0.0005^a^	
< 3	95 (57.58)	50 (30.30)	45 (27.27)			Reference
≥ 3	70 (42.42)	18 (10.91)	52 (31.52)		0.0007^b^	3.210 (1.642–6.276)
Lymph node status				11.334	0.0008^a^	
Negative	109 (66.06)	55 (32.73)	54 (32.73)			Reference
Positive	56 (33.94)	13 (7.88)	43 (26.06)		0.0010^b^	3.369 (1.631–6.957)
ER				3.137	0.0765^a^	
Negative	54 (32.73)	17 (10.30)	37 (22.42)			Reference
Positive	111 (67.27)	51 (30.91)	60 (36.36)		0.0783^b^	0.541 (0.272–1.072)
PR				1.203	0.2726^a^	
Negative	54 (32.73)	19 (11.52)	35 (21.21)			Reference
Positive	111 (67.27)	49 (29.70)	62 (37.58)		0.2737^b^	0.687 (0.351–1.346)
HER2				0.232	0.6298^a^	
Negative	138 (83.64)	58 (35.15)	80 (48.48)			Reference
Positive	27 (16.36)	10 (6.06)	17 (10.30)		0.6302^b^	1.232 (0.526–2.887)
Ki-67 index (%)				7.466	0.0063^a^	
< 20	34 (20.61)	21 (12.73)	13 (7.88)			Reference
≥ 20	131 (79.39)	47 (28.48)	84 (50.91)		0.0076^b^	2.887 (1.326–6.288)
Molecular subtype				2.570	0.4628^a^	
Luminal A	23 (13.94)	12 (7.27)	11 (6.67)			Reference
Luminal B	81 (49.09)	35 (21.21)	46 (27.88)		0.4470^b^	1.434 (0.566–3.629)
HER2	27 (16.36)	10 (6.06)	17 (10.30)		0.2845^b^	1.855 (0.598–5.748)
TNBC	34 (20.61)	11 (6.67)	23 (13.94)		0.1377^b^	2.281 (0.768–6.776)
Histological grade				8.282	0.0040^a^	
I–II	127 (76.97)	60 (36.36)	67 (40.61)			Reference
III	38 (23.03)	8 (4.85)	30 (18.18)		0.0054^b^	3.358 (1.429–7.890)
TNM stage				23.606	< 0.0001^a^	
I	37 (22.42)	28 (16.97)	9 (16.97)			Reference
II	109 (66.06)	35 (21.21)	74 (44.85)		< 0.0001^b^	6.489 (2.767–15.217)
III	19 (11.52)	5 (3.03)	14 (8.48)		0.0005^b^	9.333 (2.647–32.915)

^a^*p*-value—came from Pearson Chi-square tests or Fisher’s exact test

^b^*p*-value—came from Logistic regression analyses

**Fig. 3:**
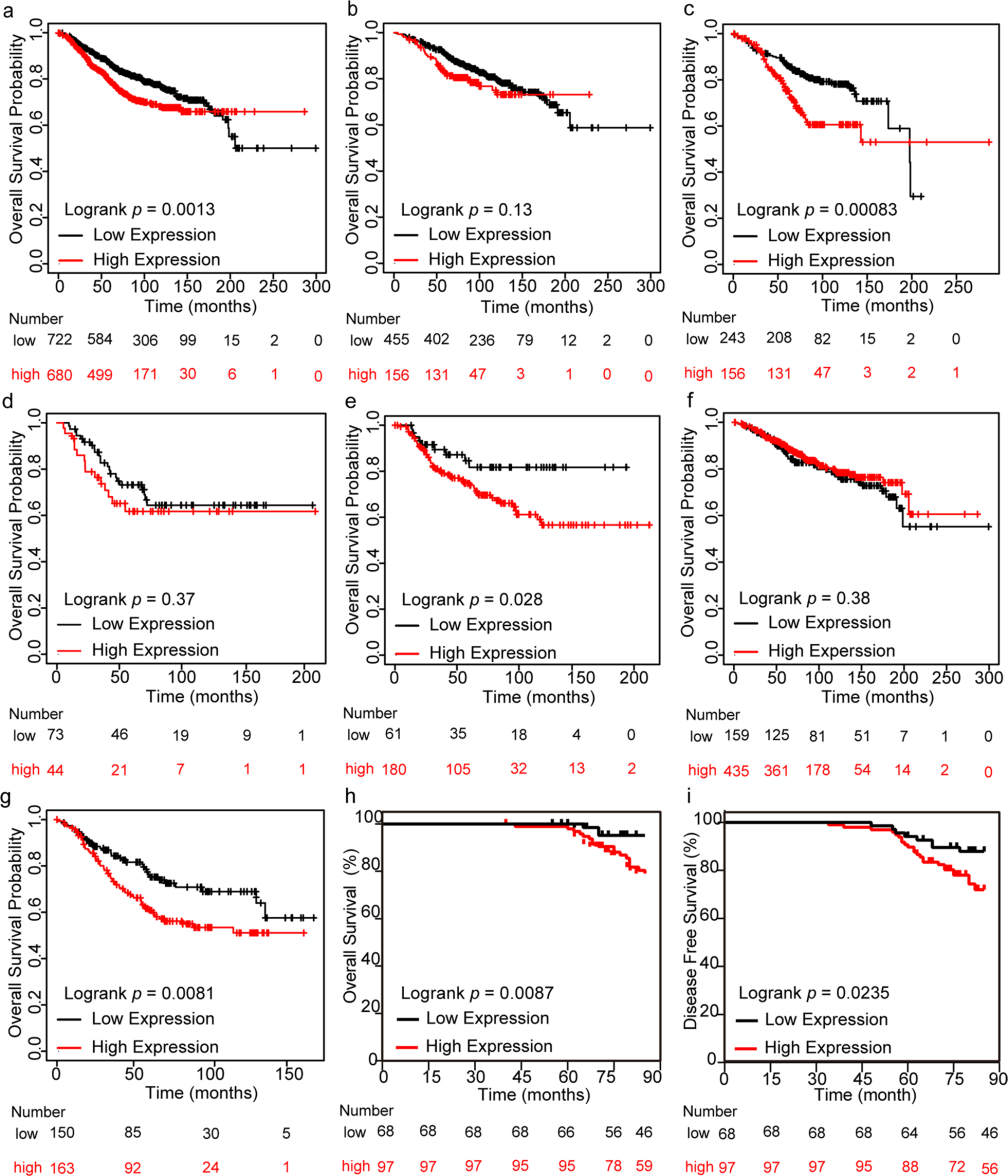
Association between DDX27 expression and prognosis. **a** High DDX27 expression was related to shorter OS in all breast cancer patients in Kaplan–Meier plotter (*p* = 0.0013). **b** High DDX27 expression was related to shorter OS in Luminal A breast cancer patients in Kaplan–Meier plotter (*p* = 0.13). **c** High DDX27 expression was related to shorter OS in Luminal B breast cancer patients in Kaplan–Meier plotter (*p* = 0.00083). **d** High DDX27 expression was related to shorter OS in HER2 breast cancer patients in Kaplan–Meier plotter (*p* = 0.37). **e** High DDX27 expression was related to shorter OS in Basal-like breast cancer patients in Kaplan–Meier plotter (*p* = 0.028). **f** High DDX27 expression was not related to shorter OS in lymph node negative breast cancer patients in Kaplan–Meier plotter (*p* = 0.38). **g** High DDX27 expression was related to shorter OS in lymph node positive breast cancer patients in Kaplan–Meier plotter (*p* = 0.0081). **h** High expression of DDX27 was significantly associated with a shorter OS in 165 patients (*p* = 0.0087). **i** High expression of DDX27 was significantly associated with a shorter DFS in 165 patients (*p* = 0.0235)

Bioinformatic analysis according to Kaplan–Meier plotter and Log‐Rank test proved that higher expression of DDX27 was significantly relevant to worse OS in breast cancer (*p* = 0.0013, Fig. 3a). Further, we analyzed the implication of DDX27 on the prognosis in terms of different molecular subtypes and found that the shorter OS associated with DDX27 was especially showed in Luminal B breast cancer (*p* = 0.00083) and Basal-like breast cancer (*p* = 0.028) (Fig. 3b–e). Moreover, we also found that higher expression of DDX27 could lead to a shorter OS in patients with lymph nodes metastasis compared to the patients without metastasis (*p* = 0.38 in lymph node negative and *p* = 0.0081 in lymph node positive, Fig. 3f, g).

Our analysis of 165 breast cancer patients had a consistent result with the bioinformatic analysis on prognosis. The Kaplan–Meier analysis and Log-Rank test suggested that DDX27 was positively and closely connected with shorter OS and DFS respectively (*p* = 0.0087 for OS and *p* = 0.0235 for DFS, Fig. 3h, i). Furthermore, we found that DDX27 expression (*p* = 0.017), tumor size (*p* = 0.0004), lymph node status (*p* < 0.0001), histological grade (*p* = 0.0004) and TNM stage (*p* < 0.0001) were in connection with worse OS in univariate Cox regression, and multivariate analysis showed the independent factors contained tumor size (*p* = 0.032), lymph node status (*p* = 0.013) and histological grade (*p* = 0.015) (Table 2). DDX27 expression (*p* = 0.0289), tumor size (*p* = 0.0004), lymph node status (*p* < 0.0001) and TNM stage (*p* = 0.0001) were correlated to worse DFS in univariate analysis. Multivariate analysis proved that tumor size (*p* = 0.032) and lymph node status (*p* = 0.018) were related to DFS independently (Table 3). Therefore, high expression of DDX27 was closely connected with different clinicopathological factors and resulted in a worse prognosis in breast cancer.

**Table 2:** Univariate and multivariate Cox regression analysis of DDX27 expression with regard to OS in 165 breast cancer patients

Characteristics	Univariate analysis		Multivariate analysis	
	HR(95% CI)	*p*-value	HR(95% CI)	*p*-value
DDX27	4.437 (1.307–15.064)	0.017	1.363 (0.358–5.189)	0.650
Age	0.551 (0.162–1.871)	0.339		
Tumor size	9.220 (2.716–31.307)	0.0004	4.178 (1.127–15.488)	0.032
Lymph node status	7.613 (2.786–20.807)	< 0.0001	4.339 (1.364–13.804)	0.013
ER	1.002 (0.404–2.483)	0.996		
PR	1.025 (0.414–2.539)	0.958		
HER2	1.603 (0.587–4.378)	0.357		
Ki-67 index (%)	1.640 (0.483–5.568)	0.428		
Histological grade	4.805 (2.024–11.405)	0.0004	3.093 (1.243–7.698)	0.015
TNM stage	6.045 (2.498–14.626)	< 0.0001	1.296 (0.475–3.533)	0.613

**Table 3:** Univariate and multivariate Cox regression analysis of DDX27 expression with regard to DFS in 165 breast cancer patients

Characteristics	Univariate analysis		Multivariate analysis	
	HR(95% CI)	*p*-value	HR(95% CI)	*p*-value
DDX27	2.420 (1.095–5.346)	0.0289	1.336 (0.575–3.108)	0.501
Age	0.692 (0.287–1.672)	0.414		
Tumor size	3.773 (1.804–7.893)	0.0004	2.426 (1.081–5.444)	0.032
Lymph node status	4.319 (2.135–8.736)	< 0.0001	2.736 (1.190–6.290)	0.018
ER	1.040 (0.507–2.134)	0.914		
PR	1.429 (0.667–3.061)	0.359		
HER2	1.327 (0.578–3.049)	0.504		
Ki-67 index (%)	2.033 (0.716–5.771)	0.183		
Histological grade	1.736 (0.846–3.563)	0.132		
TNM stage	4.181 (1.996–8.785)	0.0001	1.434 (0.602–3.421)	0.416

### DDX27 promote breast cancer by enhancing stem cell-like properties

**Fig. 4:**
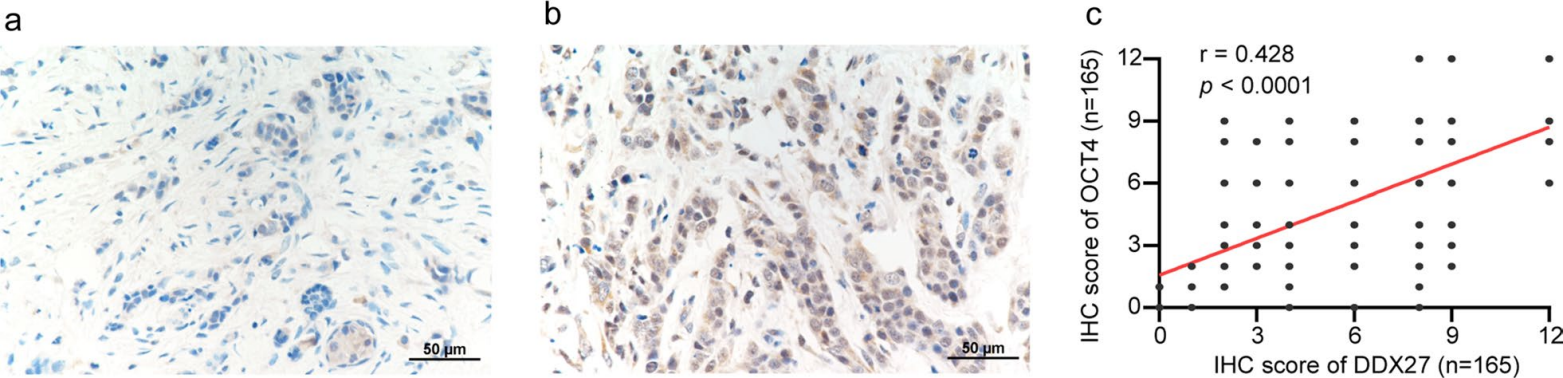
Relationship between DDX27 and OCT4 by IHC staining. **a** Low expression of OCT4. **b** High expression of OCT4. **c** Correlation between DDX27 and OCT4 expression by IHC staining (Spearman *p* < 0.0001, r = 0.428)

**Fig. 5:**
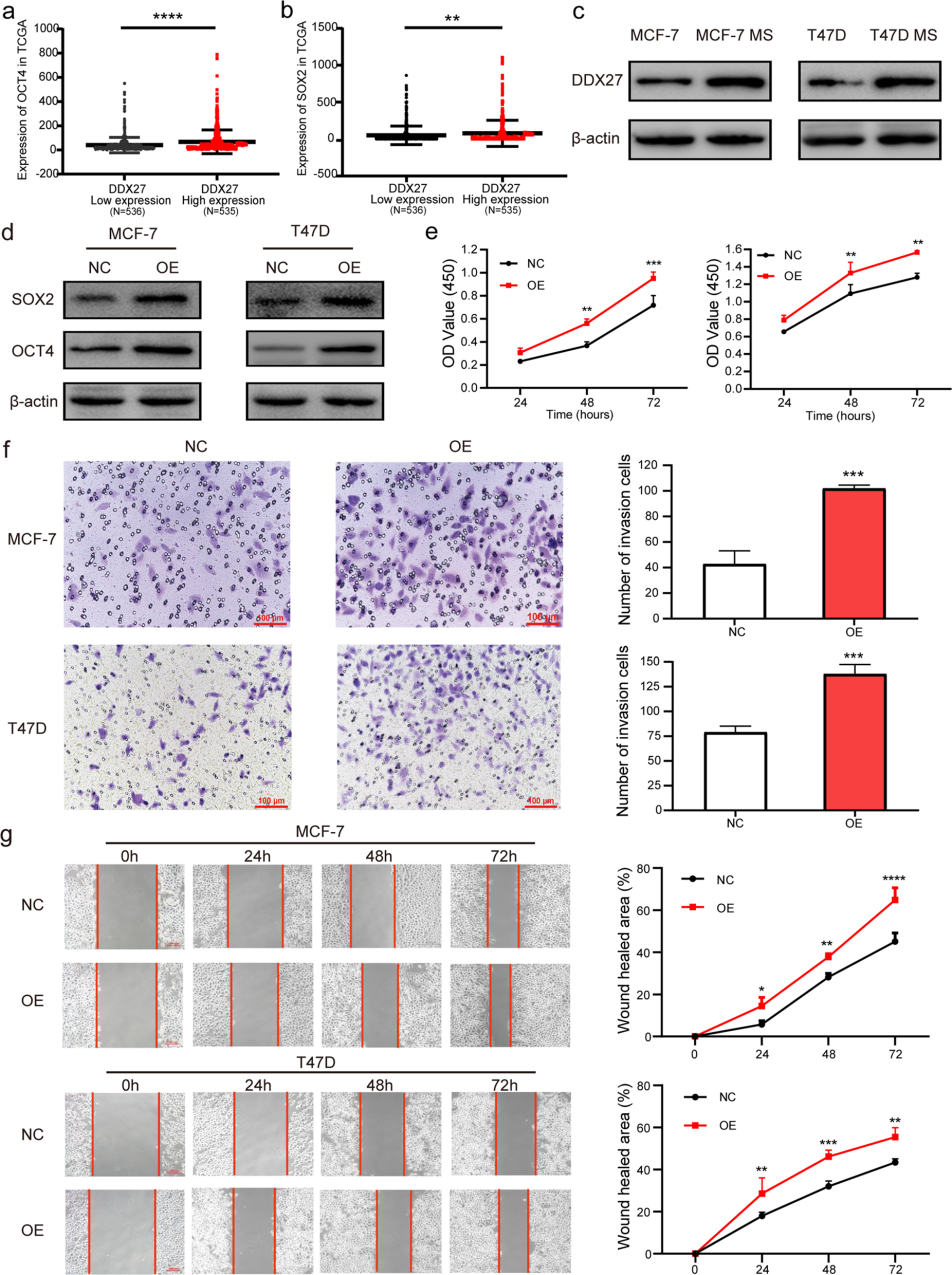
DDX27 promotes the stem cell-like properties of breast cancer cells. **a**, **b** High expression of DDX27 was positively related to OCT4 and SOX2. **c** DDX27 was significantly high expressed in MCF-7 MS and T47D MS. **d** SOX2 and OCT4 were up-regulated in DDX27 over-expressed cells by western blot. **e** Proliferation abilities of breast cancer cells by CCK-8 assay. **f** Migration abilities of breast cancer cells by the Transwell assay. **g** Migration abilities of breast cancer cells by the wound-healing assay. *p < 0.05, **p < 0.01, ***p < 0.001, and ****p < 0.0001

DDX27 was proved to act as a promoter in colorectal cancer by affecting the stem cell-like characteristics [22]. Aimed to excavate the influence of DDX27 on stem cell-like properties in breast cancer, we analyzed the relevance between the expression of DDX27 and stemness biomarkers in TCGA-BRCA database. Results proved that DDX27 had positive correlation with the expression of OCT4 (*p* < 0.0001) and SOX2 (*p* = 0.0032) (Fig. 5a, b). OCT4 is a classical biomarker correlated to breast cancer stem cells and has the expressed location in cell nucleus. In order to explore whether DDX27 affects the stem cell-like characteristics in breast cancer, we analyzed the association between DDX27 and OCT4 in 165 breast cancer patients by IHC staining and confirmed that DDX27 was positively related to the expression level of OCT4 (*p* < 0.0001, r = 0.428, Fig. 4a–c). Since our research group has proven technology on inducing and cultivating of MCF-7 MS and T47D MS, MCF-7 and T47D changed its morphology into mammospheres after inducing and grew rapidly 7–8 days later [24]. According to previous research of our group, the expression of stemness biomarkers and stem cell-like characteristics were obviously enhanced in MCF-7 MS and T47D MS [24, 25]. In our research, we confirmed that DDX27 was significantly high-expressed in MCF-7 MS and T47D MS compared to MCF-7 and T47D by western blot (Fig. 5c). To further elucidate DDX27 as a breast cancer stem cell biotarget, we transfected over-expression and negative control plasmid into MCF-7 and T47D cells and found that SOX2 and OCT4 were up-regulated in DDX27 overexpressed cells on protein levels (Fig. 5d).

We also investigated whether DDX27 could impact on proliferation and migration in breast cancer cells. Results of CCK-8 assay proved that overexpression of DDX27 could improve the proliferation ability in MCF-7 and T47D cells (Fig. 5e). Transwell assay confirmed the improvement of migration ability in DDX27 overexpressed cells (Fig. 5f), and the wound-healing assay proved that cells with overexpressed DDX27 could significantly increase the wound-healing ability (Fig. 5g). All of our results suggested that DDX27 could enhance the stem cell-like properties in breast cancer.

### DDX27-related signaling pathways

**Fig. 6:**
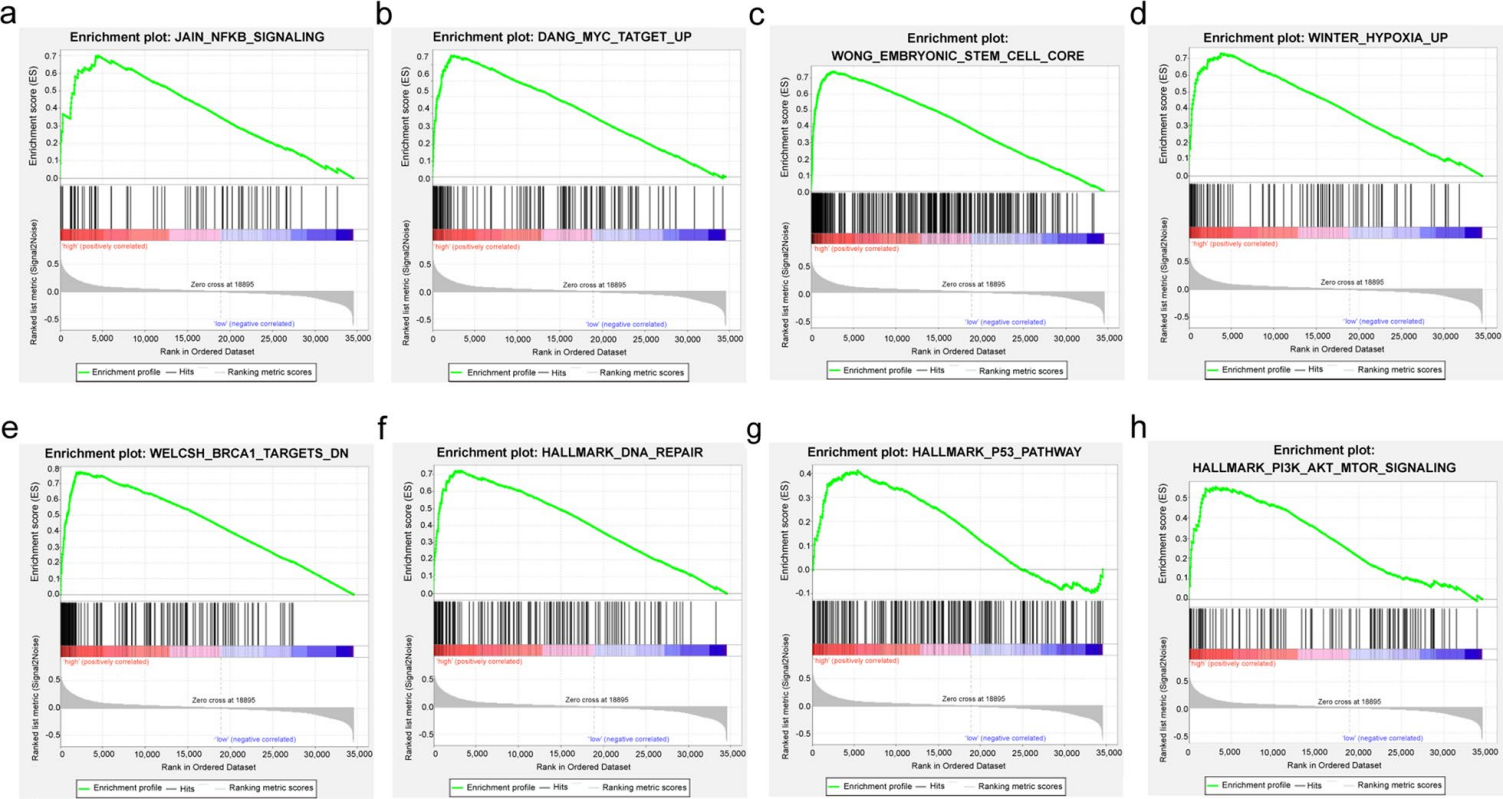
DDX27-related signaling pathways from Gene Set Enrichment Analysis (GSEA). **a** NF-κB pathway; **b** MYC targets; **c** Embryonic stem cell core; **d** Hypoxia; **e** BRCA1 targets; **f** DNA repair; **g** p53 pathway; **h** PI3K-AKT-mTOR pathway

GSEA was carried out to investigate the potential molecular mechanisms related to DDX27 in breast cancer based on the integrated data from TCGA-BRCA. The results of GSEA indicated that high expression of DDX27 was positively related to NF-κB signaling, embryonic stem cell core, DNA repair, p53 pathway, PI3K-AKT-mTOR signaling and the genes which can upregulate MYC targets, hypoxia related genes and downregulate BRCA1 targets (Fig. 6a–h, Table 4). Core enrichment genes of each signaling pathway were shown in Additional file 2: Table S1. This analysis indicated that DDX27 might participate in various signaling pathways in breast cancer.

**Table 4:** Gene set enriched with DDX27 high expression

MsigDB collection	Gene set name	NES	NOM *p*-val	FDR q-val
c2.cgp.v6.2.symbols.gmt	JAIN_NFKB_SIGNALING	2.689	0.000	0.000
	DANG_MYC_TARGETS_UP	2.655	0.000	0.000
	WONG_EMBRYONIC_STEM_CELL_CORE	2.583	0.000	0.000
	WINTER_HYPOXIA_UP	2.711	0.000	0.000
	WELCSH_BRCA1_TARGETS_DN	2.798	0.000	0.000
h.all.v6.0.symbols.gmt	HALLMARK_DNA_REPAIR	2.683	0.000	0.000
	HALLMARK_P53_PATHWAY	1.778	0.000	0.000
	HALLMARK_PI3K_AKT_MTOR_SIGNALING	2.199	0.000	0.000

*NES* normalized enrichment score, *NOM* nominal, *FDR* false discovery rate

## Discussion

Improving prognosis is always a pursuit of cancer therapy. Treatment strategies based on breast cancer molecular types has brought the effective improvement of diagnosis and therapy with better prognosis, which indicates that screening effective biomarkers is always of great significance. As we mentioned before, DDX27 was reported as a promoter and a biomarker with worse prognosis in hepatocellular carcinoma and gastrointestinal cancer [11–13]. Therefore, we analyzed the expression level of DDX27 and its influence on breast cancer in this research.

Results of our study confirmed that DDX27 was significantly high-expressed in both bioinformatics analysis and breast cancer samples. Analysis based on DDX27 expression and clinicopathological factors proved that high expression of DDX27 was closely associated with larger tumor size, positive lymph nodes, higher ki-67, higher histological grade and later TNM stage. Analysis of CPTAC database showed that high expression of DDX27 was closely related to the molecular subtypes but we didn’t get the same results in the 165 breast cancer patients. Reason for the different results might because the data from CPTAC database totally contained 125 breast cancer patients from different races (Additional file 1: Figure S1c), while our study contained 165 breast cancer patients came from China. Therefore, the influence of DDX27 expression on molecular subtypes in breast cancer should be explored in a larger size of data in the future work.

A consistent conclusion that DDX27 was positively related to a worse prognosis was obtained on Kaplan–Meier plotter and breast cancer patients. Univariate analysis of OS and DFS in 165 breast cancer patients showed that higher DDX27 expression, larger tumor size, positive lymph nodes and later TNM stage were correlated to worse prognosis, while the larger tumor size and positive lymph nodes were related to a worse prognosis in multivariate analysis. Based on our research, DDX27 is suggested to be a potential biomarker related to prognosis. Since we only analyzed 165 patients, multivariate analysis didn’t get a positive result on DDX27 expression. In future, studies including more patients are required to investigate the effect of DDX27 in breast cancer.

Cancer stem cells act as a crucial part in the processes of tumorigenesis, progress, migration, and therapeutic drug resistance [14–16]. DDX27 was proved to act as a promoter in tumorigenesis by impacting stem cell-like characteristics in colorectal cancer [22]. With the inspiration, we explored whether DDX27 could enhance the stem cell-like properties in breast cancer. In this research, we firstly accessed the correction between DDX27 expression and stemness biomarkers. Analysis based on TCGA-BRCA and breast cancer samples confirmed that DDX27 had a positive connection with the expression level of OCT4 significantly. Increased expression of stemness biomarkers and the enhanced abilities of proliferation and migration were shown in DDX27 over-expressed MCF-7 and T47D cells, which indicated that DDX27 could enhance the stem cell-like properties in breast cancer.

The processes of tumorigenesis, development and invasion are regulated by multifarious signaling pathways. In this study, we analyzed the potential molecular mechanism related to DDX27 in breast cancer by GSEA. NF-κB pathway has a great influence on regulating the biological behaviors of breast cancer stem cells [26, 27]. Enhancement of NF-κB pathway perhaps indicates that DDX27 might facilitate the processes of Epithelial–Mesenchymal Transition (EMT) and have effects on the therapeutic drug resistance in breast cancer [28]. Interestingly, DDX27 was reported to increase cancer progress and metastasis by regulating NF-κB in colorectal cancer [12]. MYC is known as a crucial factor in cancer stem cells. Overexpression of MYC might induce the ability of self-renewal and multidirectional differentiation in breast cancer stem cells [29]. Connection between DDX27 expression and hypoxia pathway might relate to oxidative stress responses in tumorigenesis in breast cancer. DDX27 correlated to BRCA1 targets and DNA repair, which suggested that DDX27 might take part in the process of genetic mutation. Moreover, DDX27 might regulate the proliferation and migration in breast cancer development on the basis of p53 pathway. We also found that DDX27 was relevant to PI3K-AKT-mTOR pathway, which could act as a therapeutic targe and might provide new ideas for treatment of breast cancer. To sum up, DDX27 might regulate breast cancer via multiple pathways, but the precise regulation mechanism is still unclear. In future research, it is necessary to carry out the mechanistic investigations to explore the influence of DDX27 on the biological properties in breast cancer.

## Conclusions

In conclusion, DDX27 was significantly high-expressed in cancer contrasted to the normal breast tissue. According to our research, the expression level of DDX27 had a closely association with larger tumor, positive lymph nodes, higher histological grade, later TNM stage and a worse prognosis. Our study suggested that DDX27 promoted the evaluation of breast cancer by influencing stem cell-like properties and the exploration of DDX27-related signaling pathways had the potential significance on figuring out the molecular mechanism of breast cancer development. In short, DDX27 is bound up with the poor prognosis by enhancing stem cell-like properties and may become a potential therapeutic target in breast cancer.

## Supplementary Information

**Additional file 1: Figure S1. Protein expression of DDX27 in breast cancer based on CPTAC database.** (a) Relationship of DDX27 expression and clinical stage in CPTAC database. (b) Relationship of DDX27 expression and molecular subtypes in breast cancer based on CPTAC database. (c) Relationship of DDX27 expression and patients’ races in CPTAC database. *p < 0.05, **p < 0.01, ***p < 0.001, and ****p < 0.0001.

**Additional file 2: Table S1.** Core enrichment genes in DDX27 related pathways.

## Data Availability

The datasets analyzed for this study can be found in the TCGA repository (https://cancergenome.nih.gov/).

## Abbreviations

DDX27: DEAD-box helicase 27
IHC: Immunohistochemistry
GSEA: Gene set enrichment analysis
OS: Overall survival
DFS: Disease free survival
TCGA-BRCA: The Cancer Genome Atlas-Breast Cancer
CPTAC: Clinical Proteomic Tumor Analysis Consortium
MsigDB: Molecular Signatures Database
ER: Estrogen receptor
PR: Progesterone receptor
HER2: Human epidermal growth factor receptor 2
PBS: Phosphate-buffered saline
BSA: Bovine serum albumin
DAB: Diaminobenzidine
CCK-8: Cell Counting Kit-8
NES: Normalized enrichment score
EMT: Epithelial–mesenchymal transition
