# Supplementary figures and images for: DEAD-box helicase 27 enhances stem cell-like properties with poor prognosis in breast cancer
Source: J Transl Med. 2021 Aug 6;19:334. doi: 10.1186/s12967-021-03011-0 (PMC8344201; doi:10.1186/s12967-021-03011-0)

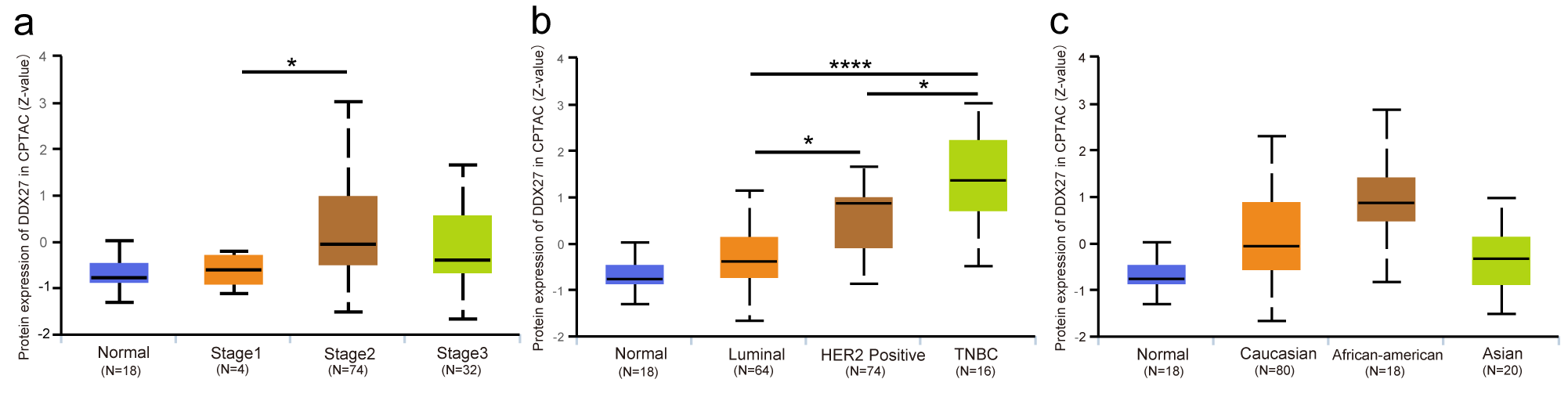

Supplement: Supplementary file 1 — Additional file 1: Figure S1. Protein expression of DDX27 in breast cancer based on CPTAC database. (a) Relationship of DDX27 expression and clinical stage in CPTAC database. (b) Relationship of DDX27 expression and molecular subtypes in breast cancer based on CPTAC database. (c) Relationship of DDX27 expression and patients’ races in CPTAC database. *p < 0.05, **p < 0.01, ***p < 0.001, and ****p < 0.0001. [file 12967_2021_3011_MOESM1_ESM.tif]
