# Supplementary material for: DEAD-box helicase 27 enhances stem cell-like properties with poor prognosis in breast cancer
Source: J Transl Med. 2021 Aug 6;19:334. doi: 10.1186/s12967-021-03011-0 (PMC8344201; doi:10.1186/s12967-021-03011-0)
Supplement: Supplementary file 2 — Additional file 2: Table S1. Core enrichment genes in DDX27 related pathways. [file 12967_2021_3011_MOESM2_ESM.docx]

**Additional file 2: Table S1** Core enrichment genes in DDX27 related pathways

| MsigDB collection | Gene set name | Core enrichment gene |
| --- | --- | --- |
| c2.cgp.v6.2.symbols.gmt | JAIN_NFKB_SIGNALING | TPD52L2, ALG3, RCC1, NOC2L, TRMT1, CLUH, CBX3, GBA, PSMA2, PMVK, LMAN2, TICAM1, STK4, DECR1, DCTN1, DDX42, LIG3, CLNS1A, SLC29A1, VEGFA, ORC5, BNIP3, SYMPK, PLAU, PSPH, PEX14, ZNF195, MED1 |
|  | DANG_MYC_TARGETS_UP | E2F1, SNRPB, RCC1, CDC25C, PCNA, DKC1, HSPE1, CCT5, CDC25A, HMGA1, SNRPD3, BAX, POLD2, TOP1, APEX1, CKS2, SRSF7, HSPD1, CAD, TK1, MTHFD1, TXN, GNL3, CDK4, SRSF1, SMN1, IRF9, NUP155, HSPA9, CSTB, CBX3, NPM1, RPL27A, TERT, SLC25A3, LDHA, PPAT, FOSL1, RPS20, DBI, RPS17, EIF2S1, E2F3 |
|  | WONG_EMBRYONIC_STEM_CELL_CORE | PSMA7, PPM1G, CCNF, KIF22, PUS1, RUVBL1, RCC1, SNRPD1, PCNA, TIMM44, MCM7, HSPE1, CDC34, EXO1, THOC3, CDCA3, FARSA, CCT5, EIF6, CDCA5, FAM136A, RUVBL2, KIF23, PSMB6, SNRPA, MRPL11, PSMA5, PHF5A, LMNB1, RACGAP1, BAX, SSB, NDC80, VRK1, MAD2L1, MRPL15, MCM4, EXOSC7, DLGAP5, HDAC1, TCF19, APEX1, CKS2, PLK4, DNMT1, MTHFD2, UQCRH, DBF4, GEMIN2, GMNN, NDUFA9, PRIM1, MRPS18B, POLR3K, MRPL13, COX5B, SQLE, EIF3I, EEF1E1, NCAPD2, VBP1, NDUFS2, NIPSNAP1, GNL3, CDK4, EIF3K, UBE2V2, SEPHS2, SERPINH1, HSPA9, ANP32E, ERP29, NIFK, MRPS2, GLO1, SUMO1, CBX3, POLE2, PABPC1, RPL27A, SNRNP40, NDUFB8, NME4, ECHS1, ALDH7A1, RPSA, ALDOC, LSM5, DEK, LBHD1, SNX5, TCF7L1, HELLS, E2F3, UQCR11, SMC4, DLAT, DHX9, SDHC, GNPDA1 |
|  | WINTER_HYPOXIA_UP | PSMA7, TPD52L2, TMEM189, CDCA4, RUVBL2, MRPL14, TIMM23, TFAP2C, TPI1, BMS1, XPO5, GEMIN2, GSS, PSMB7, B4GALT2, PGK1, DPM2, BCAR1, HILPDA, PGAM1, CNIH4, LDHA, CA9, EIF2S1, METTL22, IGF2BP2, PFKFB4, NDUFA4L2, ANKRD9, MRPS17, VEGFA, ANGPTL4, BNIP3, C16orf74 |
|  | WELCSH_BRCA1_TARGETS_DN | RAE1, ALG3, SAFB, NSDHL, RCC1, EIF3B, , FARSA, MANF, GALK1, CYC1, USP5, BMS1, CDC37, EMG1, APEX1, EDC4, CAD, DDX11, SSRP1, SF3A3, MTHFD1, POLRMT, KDM5C, HCFC1, CDK4, HSPA1B, SRPK1, ATIC, PHKG2, PDCD11, GAL, DDT, RASSF7, PRRC2A, DNTTIP2, PABPC4, BYSL, CCNE1, CHD4, DNAJC7, CKAP4, PWP2, PPAT, AGPAT2, NUP188, EIF3E, KRT6B, BRD2, SOX4 |
| h.all.v6.0.symbols.gmt | HALLMARK_DNA_REPAIR | ADRM1, ALYREF, NELFCD, RAE1, FEN1, NELFE, PCNA, RFC4, ZNRD1, ZWINT, BCAP31, ADA, GUK1, TARBP2, SSRP1, SF3A3, NELFB, SUPT5H, UMPS, PRIM1, RFC5, NT5C, VPS28, HPRT1, POLR2D, POLR2E, ERCC3, GTF3C5, COX17, TAF10, POLR3C, MPG, ARL6IP1, EDF1, NME4, BRF2, MRPL40, GTF2F1, GTF2B, DUT, RALA, AK1, STX3, TAF13, POLR1D, GTF2H3, RPA2 |
|  | HALLMARK_P53_PATHWAY | PCNA, RHBDF2, BAX, TRIB3, ADA, ST14, TAP1, PMM1, RAB40C, EPS8L2, TPRKB, TRAFD1, NUPR1, DNTTIP2, CDKN2A, PIDD1, DDIT4, SPHK1, IFI30, RNF19B, PRMT2, CD81, RAD51C, STEAP3, AK1, TPD52L1, SOCS1, TNFSF9, RRP8, EPHX1, CTSD, MAPKAPK3, CLCA2, RPS12, HEXIM1, ALOX15B, DCXR, BAIAP2, PHLDA3, GPX2, CASP1, FAM162A, TM7SF3, IP6K2, SERTAD3 |
|  | HALLMARK_PI3K_AKT_MTOR_SIGNALING | E2F1, PFN1, PLCG1, NFKBIB, TRIB3, TRAF2, CSNK2B, PAK4, CDK4, MYD88, AKT1S1, UBE2N, SQSTM1, AKT1, STAT2, PRKAG1, RAF1, ACTR3, RAC1, TNFRSF1A, RPTOR |
